# Novel role of folate (vitamin B9) released by fermenting bacteria under Human Intestine like environment

**DOI:** 10.1038/s41598-023-47243-0

**Published:** 2023-11-18

**Authors:** Sharda Nara, Gulshan Parasher, Bansi Dhar Malhotra, Manmeet Rawat

**Affiliations:** 1 Nanobioelectronics Laboratory, Department of Biotechnology, Delhi Technological University, Delhi, 110042 India; 2 Environmental Sciences & Biomedical Metrology, CSIR-National Physical Laboratory, Dr K.S. Krishnan Road, New Delhi, 110012 India; 3 Division of Gastroenterology and Hepatology, Department of Internal Medicine, University of New Mexico School of Medicine, Albuquerque, NM 87131 USA; 4 Division of Gastroenterology and Hepatology, Department of Medicine, The Penn State University College of Medicine, Penn State University, 500 University Drive, Hershey, PA 17033 USA

**Keywords:** Biotechnology, Gastroenterology

## Abstract

The anaerobic region of the gastrointestinal (GI) tract has been replicated in the anaerobic chamber of a microbial fuel cell (MFC). Electroactive biomolecules released by the facultative anaerobes (*Providencia rettgeri*) under anoxic conditions have been studied for their potential role for redox balance. MALDI study reveals the presence of vitamin B9 (folate), 6-methylpterin, para-aminobenzoic acid (PABA) and pteroic acid called pterin pool. ATR-FTIR studies further confirm the presence of the aromatic ring and side chains of folate, 6-methylpterin and PABA groups. The photoluminescence spectra of the pool exhibit the maximum emission at 420, 425, 440, and 445 nm when excited by 310, 325, 350, and 365 nm wavelengths (day 20 sample) highlighting the presence of tunable bands. The cyclic voltammetric studies indicate the active participation of pterin pool molecules in the transfer of electrons with redox potentials at − 0.2 V and − 0.4 V for p-aminobenzoate and pterin groups, respectively. In addition, it is observed that under prolonged conditions of continuous oxidative stress (> 20 days), quinonoid tetrahydrofolate is formed, leading to temporary storage of charge. The results of the present study may potentially be useful in designing effective therapeutic strategies for the management of various GI diseases by promoting or blocking folate receptors.

## Introduction

Under normal physiological conditions, a large gradient of oxygen is known to exist in different regions of the intestine and within the epithelial cells of the large intestine^1^. The bacterial population of the gastrointestinal (GI) tract has been reported to exhibit different sensitivities to different oxygen levels, and some of the bacteria are unable to survive in the presence of oxygen^2^. In the colon portion of the intestine, oxygen levels have been recorded to be below 1 mm Hg^3^. As a result, in the human gut, the anaerobic bacteria are 100 to 1000 times more populous than aerobic bacteria^4,5^. This means that their genetic make-up allows them to thrive in these conditions, allowing for a sustained release of redox mediators, for example, vitamins which may be necessary to support the gut epithelial cell in oxygen-deprived conditions^6,7^.

In addition, the correlation of colorectal cancer has been found with intracellular redox imbalance in the body^8^ with generation of higher levels of oxidants such as of reactive oxygen species (ROS) and reactive nitrogen species (RNS) than antioxidants that can oxidize macromolecules of proteins, DNA, lipid and increased cytotoxicity^9^. In the case of colon cancer, the response of the cancer cells has been shown to be regulated by oxygen partial pressure^2^.

According to an estimate, around 1.2 million patients are diagnosed with colorectal cancer every year, and around 0.6 million die from it. The cases are reported to be higher in men as compared to women^8,10,11^. Globally, in 2018, more than 1.8 million people were diagnosed with colorectal cancer; in 2019, about 150,000 new cases were recorded, while 52,000 deaths occurred due to colorectal cancer in the United States alone^12,13^. Compared to developing countries, colorectal cancer is the leading cause of death in industrialized western countries^14^.

Current treatment strategies are based on a combination of different modalities, including chemotherapy, surgical resection, and immunomodulatory therapy. However it has been reported that the health of 40% of cancer patients eventually declines with disease recurrence, making treatment effective for less than 15% of total patients, even as they also suffered from drug resistance and other side effects. Effective target selectivity coupled with knowledge of precise scientific mechanisms may help us to treat colorectal cancer more effectively with fewer side effects^15,16^.

Several designs have previously attempted to create an oxygen gradient for the co-culture of microbes and intestinal cells to study their behavior. For example, Raehyun Kim et al. have designed a device consisting of materials with differential oxygen permeability to provide a gradient of oxygen to intestinal epithelial cells and create an oxygen-deficient lumen for co-culture of the anaerobes^17^. Pranjul Shah et al. have designed a microfluidic based model for co-culture of intestinal anaerobes and human intestinal cells^18^.

Furthermore, tumor cells show greater adaptability to persistent oxidative stress, therefore more information is needed to manipulate oxidative stress and related transcription factors to target cancer cells specifically. Otherwise, the adaptability of tumor cells makes them more drug resistant, thereby hindering cancer treatment^13^.

Thus, there is a need to study, in more detail, the gut bacterial biomolecules involved in maintaining the redox balance and the mechanism of charge transfer to prevent the generation of free radicals. This may not only prevent pathological conditions but also help to develop therapeutic options for GI diseases.

To study the nature of these biomolecules in vitro, the anodic chamber of a microbial fuel cell (MFC) can be used because it is relatively more user-friendly and allows for the release of biomolecules participating in the redox reactions. Normally, an MFC operates in the range of 0 to 20% oxygen partial pressure^19^, similar to the anoxic lumen of the intestine. In the anodic chamber of MFCs, bacteria that survive under anoxic conditions have previously reported releasing vitamins that typically act as electron acceptors and/or redox mediators in the absence of oxygen^20^. Hanne Wiessenkens et al. have studied the intestinal cell culture in an anaerobic chamber (< 0.1% O_2_). This study showed that anoxic conditions for intestinal cells reduce oxidative stress with higher oxidized glutathione levels^2^. Therefore, by providing gut-like oxygen-deprived conditions to facultative anaerobic bacteria, the released biomolecules participating in redox reactions can be isolated and studied for their potential role in preventing oxidative stress to the biological cell.

In the present study, we have taken *Providencia rettgeri*, a facultative anaerobe that is classified in the order *Enterobacteriales*^21^, a large population of which resides in the intestinal part of the human body ^22^. The fluorescence, cyclic voltammetry and circular dichroism analysis of folate molecules and folate synthesis intermediate molecules including para-aminobenzoic acid (PABA). 6-methyl pterin and pteroic acid called pterin pool released in the fuel cell anaerobic chamber has been studied to implicate the mechanism of charge transfer through these molecules to prevent oxidative stress. As it has been previously reported that with the progression of the pathological state of the tissue especially in case of neoplastic changes, the relevant physiochemical and biochemical changes occur at the tissue and cellular level could be perceived through changes in the fluorescence emission of endogenous fluorophores such as collagen, NADH and FAD. Also, the fluorescence measurement along with the measurement of redox ratio in the different tissue types, informs about the cellular metabolic rate^23–25^.

Although cyclic voltammetry and circular dichroism have different roles and purposes, they both can be applied to analyze the folate molecules in various contexts. The electrochemical activities and the redox properties of the folate and other pterin based molecules could be studied using cyclic voltammetric analysis while circular dichroism can be helpful in providing information related to the presence of chiral molecules as well as their interactions with other molecules. Both of these techniques could complement each other in finding the multifaceted properties of folate for its therapeutical use^26^.

The role of folate has previously been mainly emphasized in the synthesis of nucleic acids and amino acids^27^, while current study throws light on the new role of folate and intermediate molecules of the folate synthesis pathway including PABA, 6-methylpterin and pteroic acid secreted by fermenting bacteria in maintaining redox balance under anaerobic conditions.

PABA is an aromatic compound with a carboxylic and amino group attached to a benzene ring. It participates in the electron transfer during the synthesis of dihydropteroic acid^28^.

PABA has also been reported to be essential for various human pathogens but not indispensable for humans, thus it could be modified and used as an antimicrobial agent. Martin Krátký et al.^28^ have suggested that hybridization of PABA and aromatic aldehydes resulting in Schiff bases, this derivatization procedure actually converts the non-toxic PABA into an effective antimicrobial agent and even shown to exhibit anticancer activity towards HepG2 cell line. While 6-methylpterin, a derivative of pterin with an attached methyl group, is a heterocyclic molecule present in various biomolecules. It involves the charge transfer process in biochemical reactions where it forms a cofactor and coenzyme, such as tetrahydrobiopterin participate in many enzymatic processes including the synthesis of neurotransmitter^29^.

Another intermediate molecule is pteroic acid which is formed of a pterin ring and a moiety of PABA; its charge transfer ability mainly depends on its role as a precursor molecule in biosynthesis and other metabolic reactions^30^.

The folate molecule contains a pterin ring, a PABA moiety and a polyglutamate tail. And, it is mainly involved in the synthesis of nucleic acid and repair. Its charge transfer ability mainly occurs during the interconversion of its various forms including, dihydrofolate and tetrahydrofolate. Further, the charge transfer property of the folate is crucial when it acts as a coenzyme in case of one-carbon metabolism and participates in methyl group transfer in various biochemical reactions^31^.

Additionally, folate has been found to be correlated with the regulation of oxidative–nitrosative stress and it also acts as a scavenger of hydroxy ion radicals^32^. Also, protein acting as ROS sinker has been previously shown to decrease with low levels of folate molecules. Similarly, the mitochondrial glutathione level has been reported to be suppressed with the low levels of folate^29,32,33^.

The present study attempts to provide an understanding of the underlying mechanisms of the individual and synergistic role of these biomolecules (pterin pool) through a charge transfer to balance the oxidative stress caused by the absence of oxygen. Since oxidative stress caused by redox imbalance generates reactive oxygen species (ROS) which has been found to be significantly associated with colorectal cancer^8,13^. Therefore, the given analysis may be helpful in developing therapy to treat colorectal cancer.

## Experimental section

### Anaerobic set-up

Oxidative stress has been applied to fermenting bacteria of *Providencia rettgeri* by culturing them in an anaerobic chamber wherein oxygen was removed from the bacterial media by purging the nitrogen gas. The chamber comprises of a three-electrode system: graphite electrode as working electrode, Ag/AgCl as reference electrode, and platinum as control electrode^20^. 1 ml of *Providencia rettgeri* broth was added to 20 ml of acetate growth media. The biomolecules released in the bacterial spent media were extracted on days 2, 10, and 20 via a solvent-enriched method, following the biomolecule isolation process as given in our previous study^20^. Briefly, the bacterial spent media was centrifuged for 20 min at 8000 rpm, 4 °C. Then, the supernatant was collected and further purified by organic solvents of chloroform and methanol. Here, the chloroform and methanol were used in a 2:1 ratio (volume), and approximately its equal volume was added dropwise to the centrifuged supernatant. The solution was mixed thoroughly and left undisturbed until the aqueous and organ phases were separated. Biomolecules were gradually migrated into the organic phase because of their solvent affinity. This organic phase with biomolecules was collected gently using an auto pipet^34^.

### MALDI-TOF mass spectrometry

Detailed information regarding MALDI-TOF mass spectroscopy has been given in our previous publication^20^. MALDI-TOF mass spectrometric measurements were conducted using BRUKER Microflex (Bruker 168 Daltonics, Billerica, MA) with a 2,5-dihydroxybenzoic acid (DHB) matrix. Here, the sample of 1 µL was kept at the parafilm following addition of 1µL of DHB, the solutions were mixed thoroughly with a pipette and the mixture was kept in MSP 96 target ground steel plate. The reduced pressure was applied to dry the sample. Subsequently, the plate containing the sample was set for the mass spectroscopic measurements. Afterward, the data was obtained from 0 to 800 m/z at 60 Hz laser frequency, with 500 laser shots. The subsequent mass spectrum was binned at increments of 0.5 m/z and inspected manually for masses of interest. Masses were further imagined by false coloring using an optical image of the original sample plate.

### Photoluminescence study

The photoluminescence (PL) spectra was obtained using a spectrofluorometer (Flourolog-3, Horiba Jobin Yvon Inc.) having a 450 W xenon lamp. The excitation and emission spectra were recorded using a quartz cuvette whereas the forward geometry stage was used to remove the internal filter effect.

### Electrochemical analysis

Cyclic voltammetry (CV) was performed with top and bottom peak potentials of + 0.8 V and − 0.8 V at scan rates of 10 mV/s using Autolab AUT 85279 Potentiostat Galvanostat (Metrohm, Netherlands). NOVA 1.11 software was used to examine the recorded data while Origin 8.0 software was used for plotting the data obtained from NOVA 1.11. A three-electrode system was used to analyze the electrochemical activity in which a graphite electrode was employed as a working electrode, Ag/AgCl as the reference electrode and platinum as the counter electrode. The study was carried out to analyze the involvement of the released molecules in the redox reactions. The spent bacterial media at 2 day, 10 day and 20 day were used as an electrolyte.

### ATR-FTIR

The attenuated total reflectance-Fourier transform infrared (ATR-FTIR) spectroscopy was conducted with all samples in liquid form using PerkinElmer (USA) instrument. All spectra were measured in the transmission mode at a data acquisition rate of 5 cm^−1^ per point in the 4000 to 500 cm^−1^ range with 50 consecutive scans per sample, followed by stacked spectra analysis for relative comparison.

### UV–Vis absorption spectroscopy

The UV–Vis spectroscopic studies were performed using a UV/Vis spectrophotometer, Lambda 950, (PerkinElmer, USA) to evaluate the behavior of released biomolecules in the bacterial spent media. Purification via the solvent extraction method has been previously described^20^. Briefly the bacterial spent media was centrifuged at 8000 rpm for 20 min at 4 °C. After that the supernatant was collected and purified by organic solvents of chloroform and methanol in 2:1 ratio (volume).

### Circular dichroism

Circular Dichroism (CD) spectral analysis of 2, 10 and 20-day-old bacterial spent media was conducted using AVIV CD spectrometer (Biomedical Inc., NJ, USA). A quartz cell of 1 mm path length was used and the bandwidth was taken to be 1.0 nm. The CD spectral measurements were based on 10 consecutive scans with a multi-scan wait of 0.50 s. The response time was 1.0 s and the settling time was 0.3 s. All measurements were performed at 25 °C.

## Results

**Figure 1:**
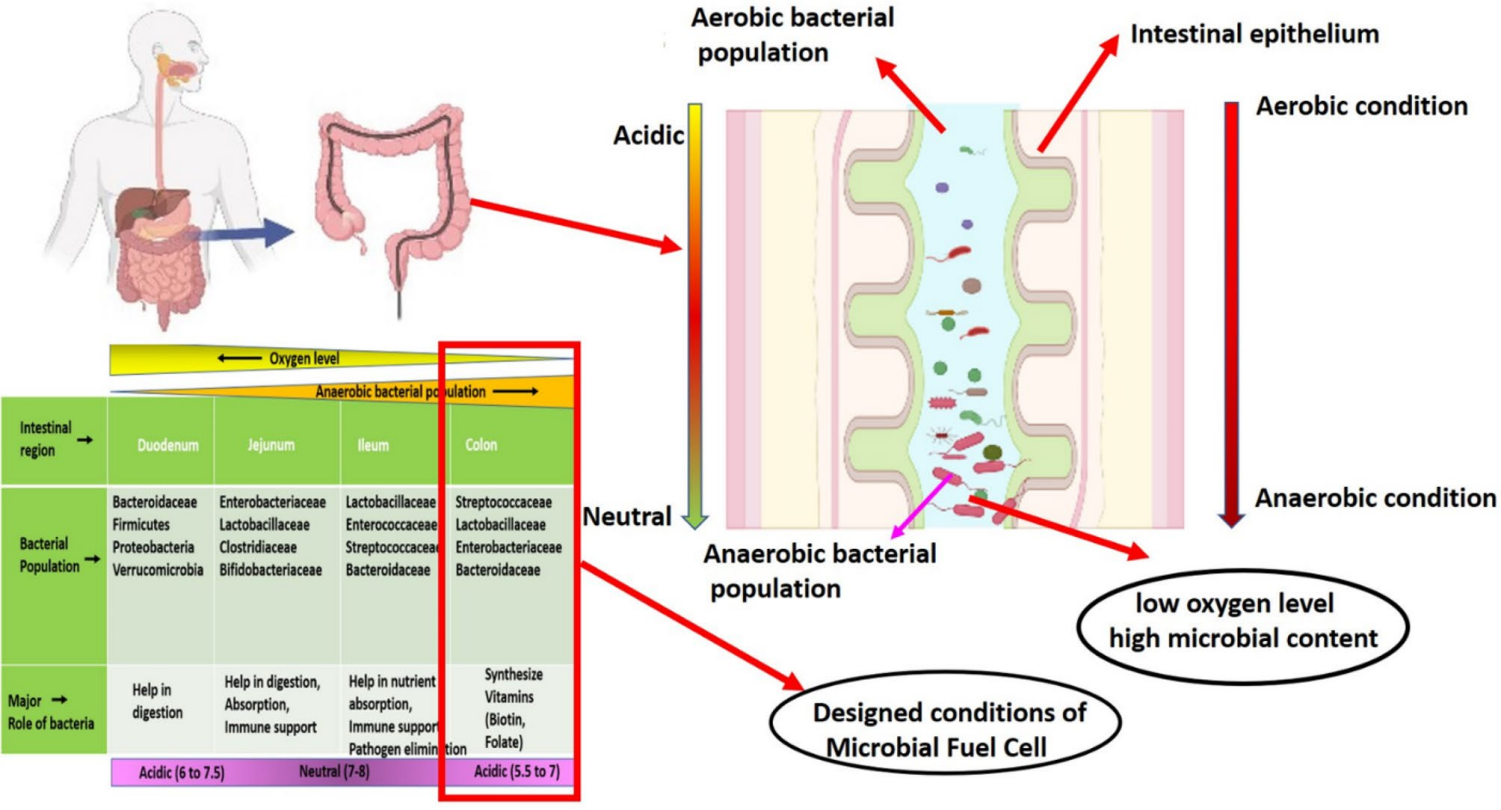
Schematic picture (image created with the help of BioRender software) showing the similarity between the conditions of low oxygen level and high microbial content in the colon region of the gastrointestinal tract and the anodic chamber of a microbial fuel cell.

**Figure 2:**
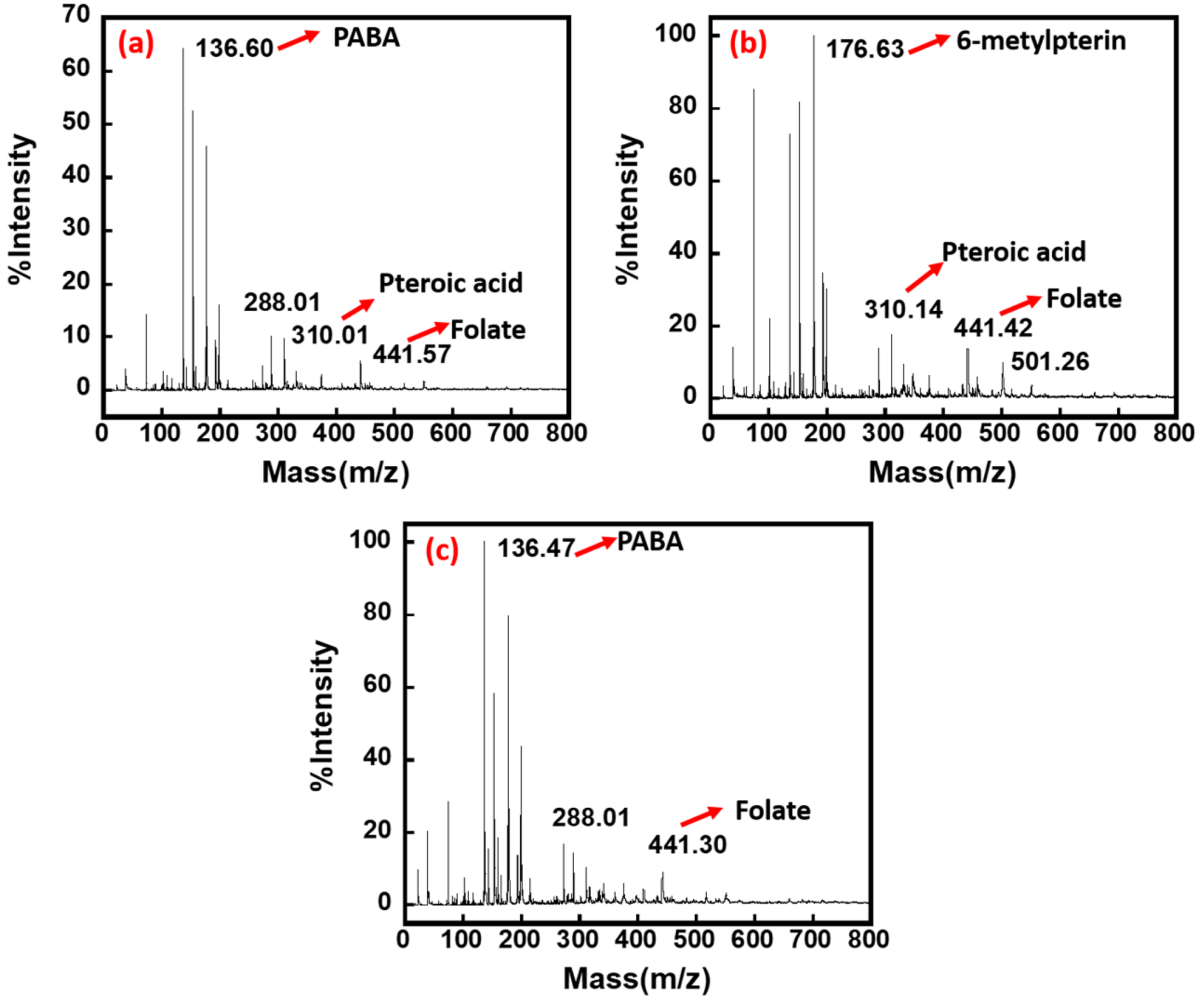
Mass spectroscopic analysis of day 2 (**a**), day 10 (**b**) and day 20 (**c**) samples of bacterial spent media, indicated the release of folate, pteroic acid, 6-methylpterin and para-aminobenzoic acid (PABA) biomolecules.

In the present study, the anaerobic condition of the colon part of our intestine (Fig. 1) was maintained in the anaerobic chamber of the MFC. The biomolecules isolated from bacterial spent media at day 2, 10 and 20 in our previous study^20^ were identified here in more detail using Mass spectroscopy (Fig. 2) to understand the ability of intermediate molecules to transfer charge and maintain redox equilibrium.

### MALDI-TOF–MS

Mass spectroscopy was conducted to identify the released biomolecules by the bacteria under anerobic conditions. Since the relative molecular mass of folate or pteroylglutamic acid (vitamin B9) is reported to be 441.4 g/mol^35,36^, hence the mass peak [M^+^ + H^+^] appeared at 441.57, 441.42 and 441.30 m/z in day 2, 10 and 20 day old spent bacterial media, respectively (Fig. 2), indicating the release of folate molecules. Further, mass peaks at 136.60 and 136.47 m/z indicated the release of PABA^37^ in day 2 and day 20 samples (Fig. 2a,c), respectively. PABA in addition to be a precursor of folate molecules, has been previously reported to reveal the antioxidant ability and has also been commercially used for UV blocking action in sunscreen^38^. It appears that PABA perhaps plays an important role in protecting the bacterial cell from the antioxidants produced in the provided anoxic condition. While the mass peak found at 176.63 m/z in day 10 (Fig. 2b) bacterial spent media indicates the release of 6-methylpterin^39^ and the mass peak seen at 310.01 and 310.14 m/z indicates the presence of the oxidized form of pteroic acid^40^ in day 2 and day 10 samples of bacterial spent media (Fig. 2a,b). Thus, the mass spectroscopy results reveal the presence of deprotonated folate and folate synthesis intermediates (PABA, 6-methylpterin, pteroic acid) called pterin pool.

As the redox imbalance can be further traced to linkage with the unregulated quantity of vitamins B, folate in particular that has been reported to reduce the oxidative stress^9,41,42^. Therefore, the release of folate molecules in all samples, indicates the molecule’s crucial role in balancing the oxidative stress by temporarily storing or transferring the charge^43^. In order to further understand the activity of the moieties and to -determine whether they are affecting each other at the molecular electronic level in oxygen-deprived conditions, circular dichroism (CD) study was conducted.

### Circular dichroism (CD) study

**Figure 3:**
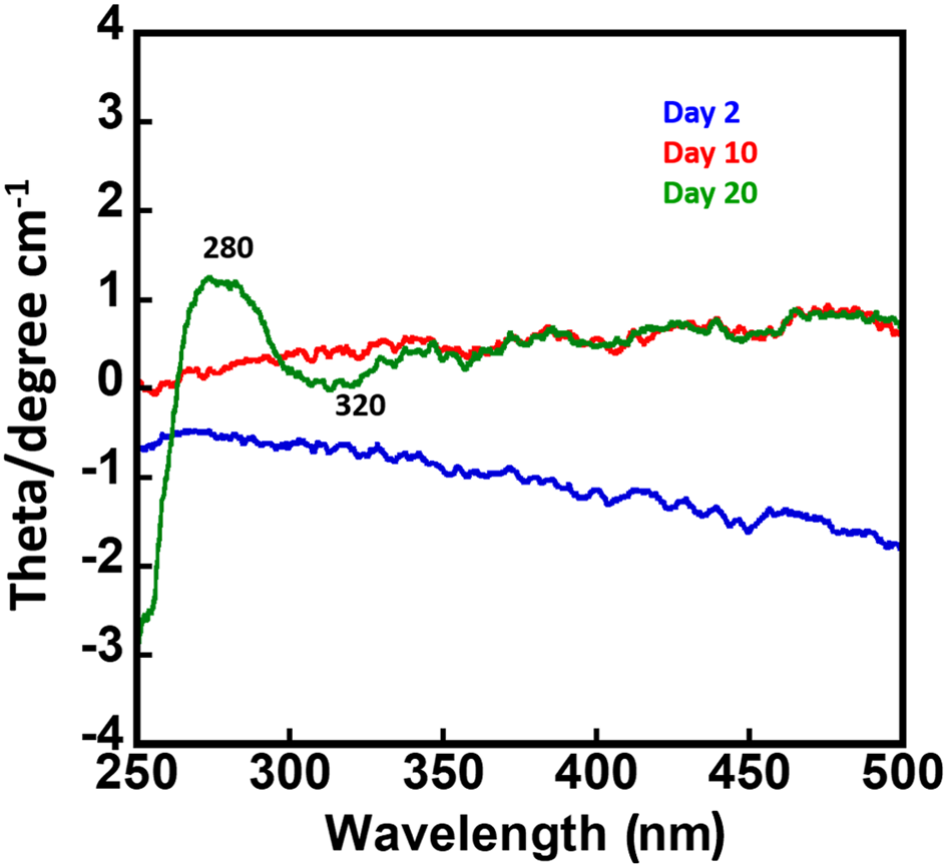
Circular Dichroism of day 2, day 10 and day 20 bacterial spent media. Spectral peaks appeared at 280 and 320 nm in day 20 sample confirm the presence of pteridine ring.

The CD spectra of the folate and pterin pool appear to be in the UV visible region. As these molecules have a pteridine ring that comprises of the conjugated double bonds, the spectral peaks found at 280 nm in the CD spectra of the pterin pool indicate characteristic bands of pteridine, which is due to the pi-electron transitions at the molecular level whereas absence of the 370 nm peak reveals that the moiety of PABA does not largely contribute to the CD signal^44,45^. Here the PABA possibly is not in a conformation that could significantly contribute to the CD signal. Furthermore, pteridine derivatives such as 6-methylpterin have been shown to exhibit CD signals at 280 nm and 320 nm (Fig. 3), which are attributed to the interaction of the pteridine ring with neighboring aromatic rings and the overall molecular conformation, respectively^46^. The CD spectral peaks are not clearly visible in day 2 and 10 samples indicating the predominant presence of precursors of PABA and pteroic acid may hinder the chiral activity of the folate and pteridine derivatives of 6-methylpterin. The release of 6-methylpterin in day 10 of bacterial growth (Fig. 2b) and its chiral activity in the CD spectra (Fig. 3) anticipate that the molecule may actively participate in molecular electronic transitions due to its favorable conformational arrangement. While the role of PABA and pteroic acid may be corelated to the free radical scavenging action to relieve the oxidative stress generated due to the anoxic condition. Molecular electronic transitions were further analyzed using UV–vis absorption spectroscopy.

### UV–Vis absorption spectroscopy

**Figure 4:**
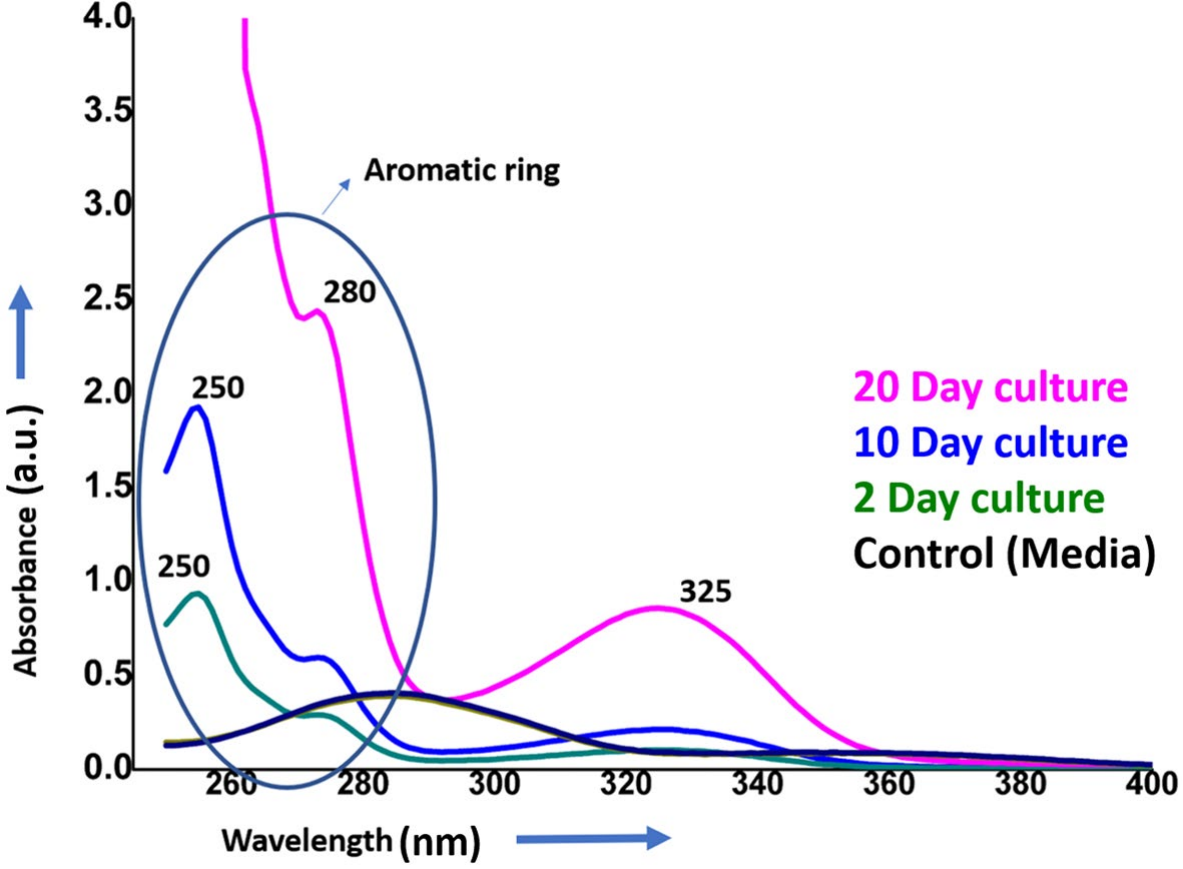
UV spectroscopy studies of folate and folate intermediates released into spent media of bacteria at 2, 10 and 20 days of bacterial growth showing spectral absorption due to para-aminobenzoic acid (PABA) and pteridine ring system.

In the UV spectroscopy study, the molecules of PABA that contribute to the absorption bands at 250 nm in the UV spectrum^47^ were predominantly found in day 2 and day 10 samples (Fig. 4). This UV spectrum band is due to the pi–pi* electronic transition of the PABA moieties^48^. Further, a spectral band seen at 325 nm which is considerably higher at day 20 (Fig. 4) attributed to the n–pi* transition of the amino group of the pteridine ring system of the 6-methylpterin and folate^49^. Although the peak at 325 nm can also be seen on day 2 and day 10, however, the intensity of the peak on day 2 is much lower, which may be due to the low expression of folate and 6-methylpterin molecules in this time period (Fig. 8). Further, at day 20, the UV spectral peak at 280 nm can be assigned to the pi–pi* transition of the pteridine ring representing the main chromophore of the folate molecule^50,51^. Overall, the UV spectral study of the molecules exhibits the peak corresponding to the pteridine ring, glutamic acid, and PABA, and no spectrum pertaining to individual molecules of the folate, 6-methylpterin and pteroic acid are seen (Fig. 4), indicating that molecules interact with each other. Previously, it has been reported that the peaks at 255 nm, 286 nm, and 366 nm are characteristic of the chromophores present in the folate molecule^52^.While in the present study, the peaks around 250, 280 and 325 nm reveal the inter and intramolecular interaction of the moieties of PABA, 6-methylpterin, pteroic acid and folate molecules to balance the absence of oxygen^43^. The functional group interactions were further investigated by an ATR-FTIR spectroscopy study.

### ATR-FTIR study

As the isolated molecules comprise of folate, 6-methylpterin, pteroic acid, and PABA which are all pterin derivatives and also have some common functional groups, there are differences in the side chains attached to the pterin ring. Consequently, their ATR-FTIR spectra may be related to the presence or absence of peaks pertaining to specific functional groups. The spectral peaks seen at 1221 cm^−1^ and 1367 cm^−1^ may be due to the C–N stretching vibrations while those at 1743 cm^−1^ and 1642 cm^−1^ may be due to the stretching vibrations of the carbonyl group (C=O) and carbon–carbon double bond (C=C) in the aromatic ring, respectively^53^.

There is an evident change in the intensity of the spectral peaks especially in the 10 day sample indicating fermi resonance in the spectral peaks at 1221 cm^−1^ and 1367 cm^−1^ as well as 1743 and 1642 cm^−1^^54^. The peak at 1435 cm^−1^ is possibly due to C=C stretching vibrations present in the aromatic ring of pterin while 1221 cm^−1^ peak could be due to C–N stretching vibrations of the functional groups present in the folate molecule^55,56^.

In the case of the day 2 sample, a sharp peak at 1011 cm^−1^ can be observed, this peak may be assigned to the C–N stretching vibration of the amino group present at the para position of the benzene ring of PABA. While no clear peak at this position has been observed at day 10 and day 20 samples, probably due to the presence of other biomolecules of the pterin pool (pteroic acid, 6-methylpterin and folate) influenced the intensity and peak position of the functional groups of PABA^57^.

**Figure 5:**
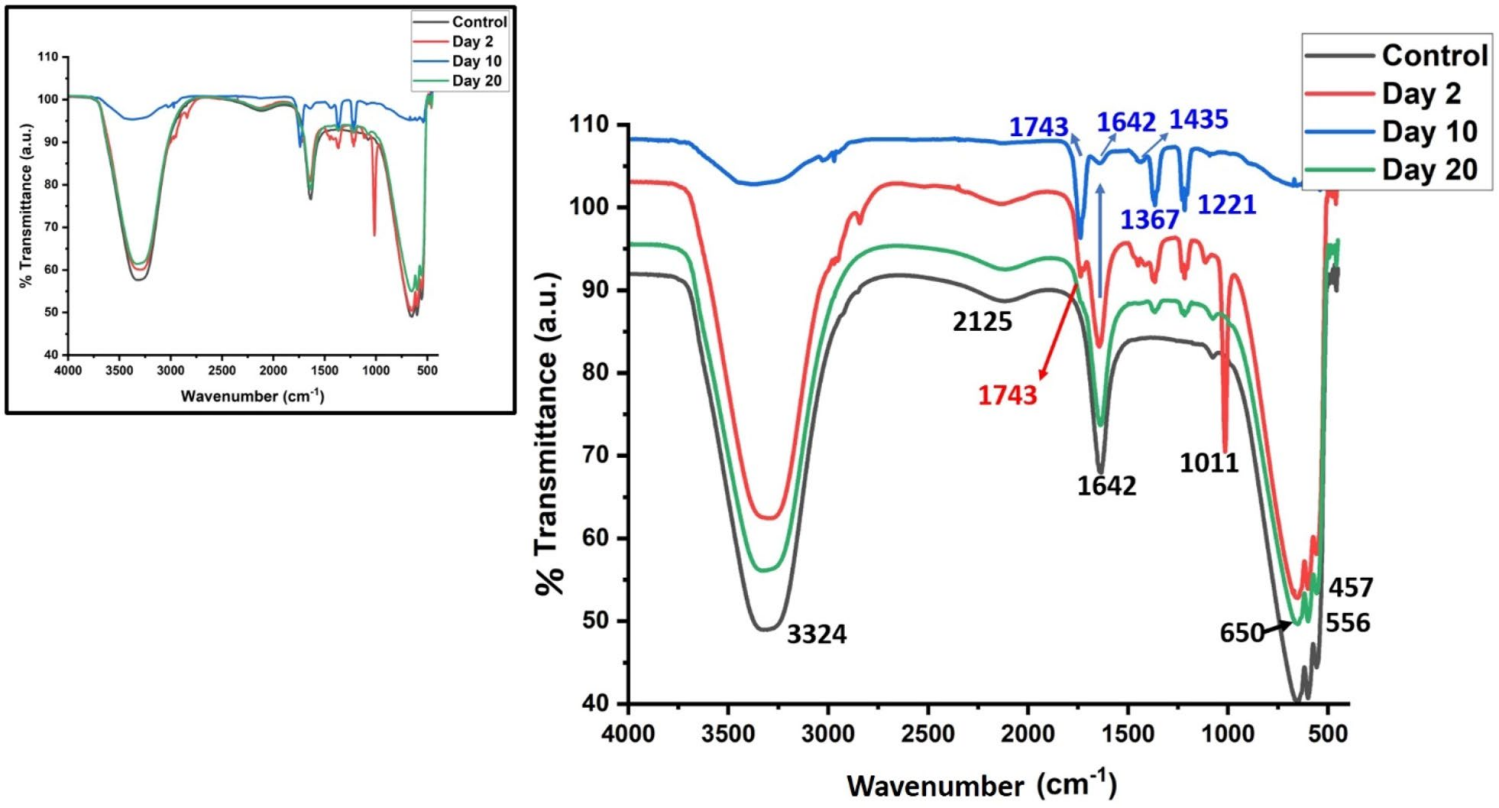
Stacked ATR-FTIR spectra of bacterial spent media at different time period of growth (Inset showing FTIR spectra of the same without stacking).

In the day 20 sample, the spectral peaks observed at 3324 cm^−1^ and 1642 cm^−1^ (Fig. 5) are due to the N–H stretching and C=O stretching vibrations of the glutamic acid side chain, respectively^58^. The spectral peaks seen at 650 cm^−1^, 556 cm^−1^, and 457 cm^−1^ in the day 20 sample (Fig. 5) may be attributed to the out-of-plane bending of the C–H bonds and N–H bonds in the pterin moiety of the molecule^59^.

In the present study the 3324 cm^−1^ peak (Fig. 5) is attributed to the N–H stretching vibration of the amide group (–CONH–) in the glutamic acid side chain of folate. This has perhaps partly overlapped due to the O–H stretching vibration present in molecules of water. And, the peak at 1642 cm^−1^ (Fig. 5) could be due to C=O stretching vibration of the amide group (–CONH–) in the glutamic acid side chain of folate^60^. The peak at 650 cm^−1^ (Fig. 5) can be associated with the out-of-plane bending of the C–H bonds in the aromatic ring of the pterin moiety of folate. While the peak at 556 cm^−1^ is perhaps due to the in-plane bending of the C-H bonds in the aromatic ring of the pterin moiety of folate and 457 cm^−1^ peak can be associated with the out-of-plane bending of the N–H bonds in the pterin moiety of folate^61–63^.

ATR-FTIR spectroscopy results show that pi–pi complexation of aromatic rings is due to charge donor and acceptor interactions which also induce the protonation process in folate molecules upon strengthening the electron-accepting ability of pterin rings. These aromatic rings have both the electron acceptors and the pi donors^64^. In order to further understand which moieties of the identified biomolecules are active in the charge transfer, the electroactivity was analyzed via cyclic voltammetry.

### Cyclic voltammetry

**Figure 6:**
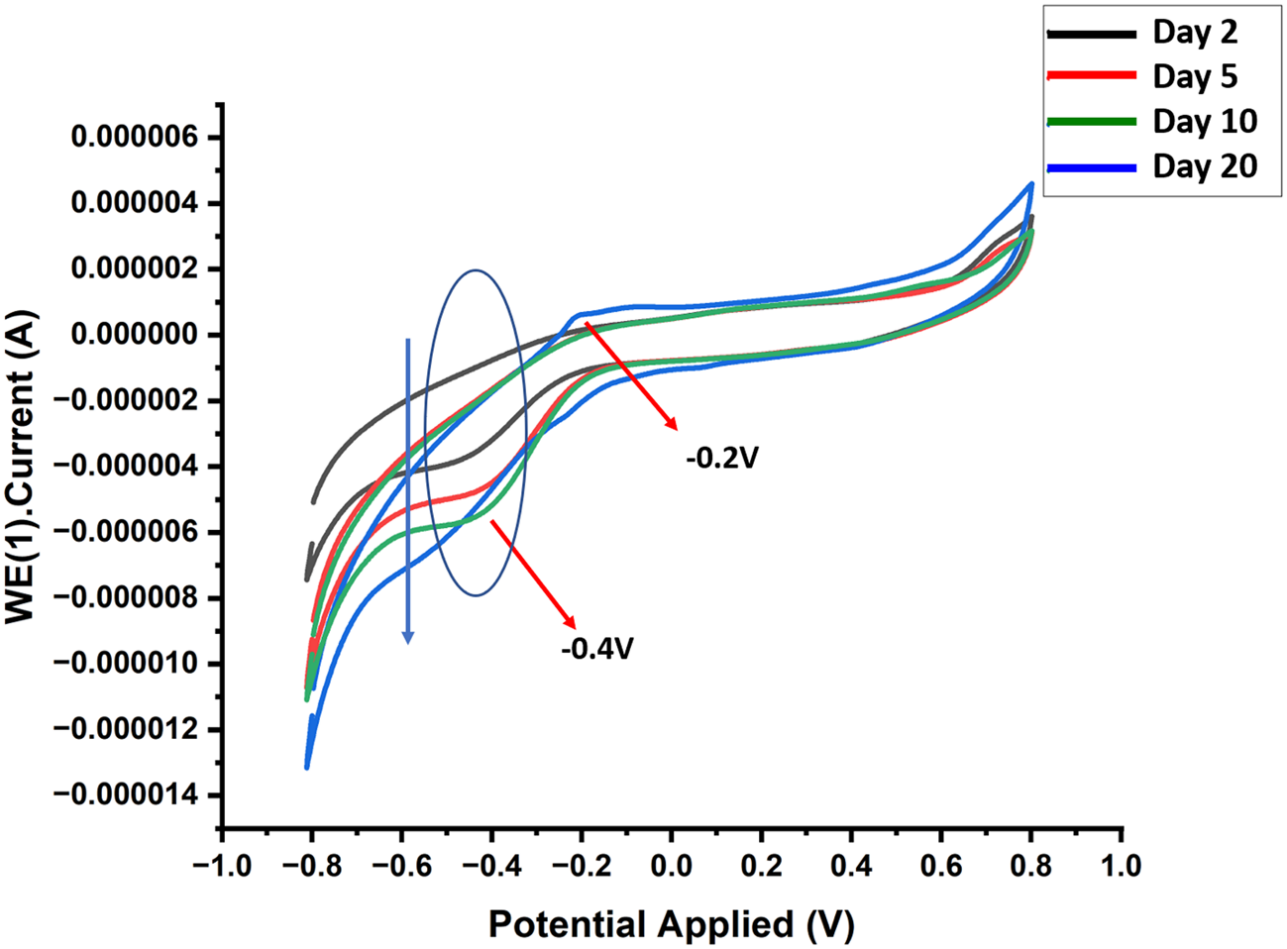
The cyclic voltammetry pattern changes with bacterial growth under anaerobic condition. The dip in curve depicting the high redox ability of the released molecules of folate and folate intermediates especially of pterin moiety and p-aminobenzoate groups.

The cyclic voltammetry study has been conducted to identify the reduction potential and may be related electrochemical reactions occurring in the anaerobic chamber. The results of cyclic voltammetry, reveal that the biomolecules released at different time periods have a difference in reduction potential. The reduction potential at day 2, 5 and 10 was observed at − 0.4 V with a shift towards more negative value indicating the greater tendencies to accept the electrons. While two clear peaks could be observed at day 20 only at − 0.2 V and − 0.4 V, it indicates that there are two electroactive moieties in these molecules, one moiety has more tendency to accept the electrons. The difference in reduction potential indicates thermodynamic favorability with the electron transfer from one species to another species each has a different reduction potential. However, as different electroactive species co-exist in a pterin pool indicates that there is a multiple electron transfer process. The two redox peaks found at − 0.4 and − 0.2 V (Fig. 6) can be associated with the reduction potentials of the pterin moiety and p-aminobenzoate groups, respectively of the pterin pool^65^. The peak observed at − 0.4 V depicts a comparatively more negative reduction potential, indicating that the pterin moiety perhaps easily gain the electrons and more easily undergo the reduction compared to the p-aminobenzoate group^66,67^.

It appears that intra-molecular electron transfer occurs with the transfer of an electron between the pterin and PABA groups of the same molecule while inter-molecular electron transfer occurs with the electron transfer from the pterin moiety of the one molecule and PABA of another molecules. These redox properties play an essential role in the cellular metabolic pathways involving the synthesis of DNA and methyl group transfer reactions.

Thus, based on the results of cyclic voltammetry, the presence of two redox-active moieties can be confirmed with different reduction potentials, and that the pterin moiety has a lower reduction potential than the PABA group. Therefore, it could be deduced that the inter and intra molecular electron transfer occurs through the PABA and pterin moieties in the absence of oxygen. Furthermore, the electrochemical behavior of the pterin pool present in a solution, depends on their relative concentrations and redox properties, determining the transfer of charge between the two molecules under the applied electrode potential. There is also a high probability of transfer of charge from 6-methylpterin to pteroic acid through an oxidation–reduction reaction, where the molecules of 6-methylpterin can easily donate the electrons to the molecules of pteroic acid. As a result, the radical anion of 6-methylpterin ions and a radical cation of pteroic acid are formed in the solution. Various factors, such as kinetics of the charge transfer and the stability of the resulting radical species could further control the efficiency of this charge transfer^68^. The probability of maintaining the redox equilibrium by charge transfer within various available energy levels has been further confirmed with the photoluminescence study.

### Photoluminescence study

**Figure 7:**
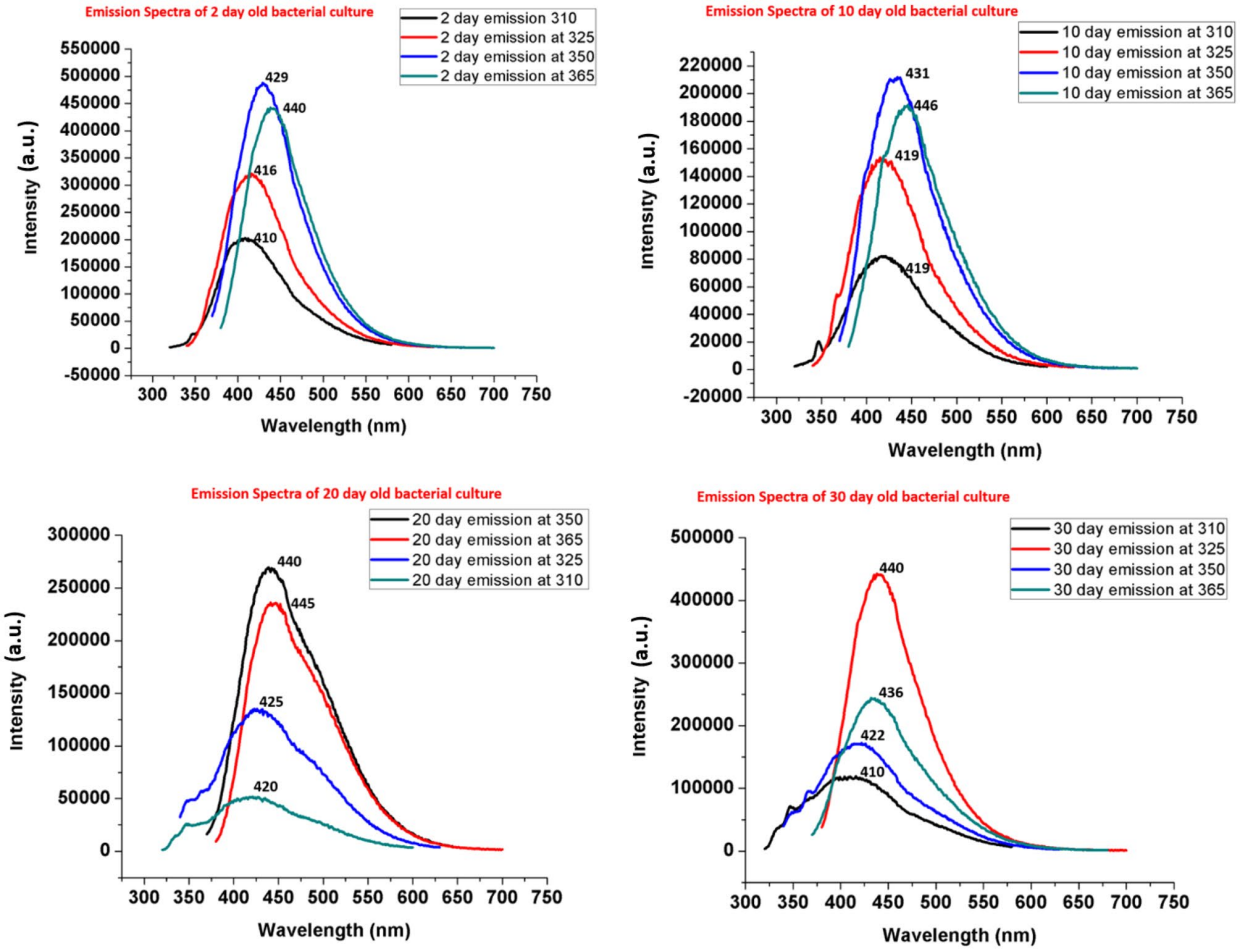
Photoluminescence emission spectra of bacterial spent media at 2 day (**a**), 10 day (**b**), 20 day (**c**) and 30 day (**d**) showing the release of folate and intermediates at different growth phase.

The photoluminescence (PL) spectra was obtained using excitation wavelengths of 310, 325, 350 and 365 nm and the emission was obtained at 410, 416, 429 and 440 nm on day 2: at 419, 419, 431 and 446 nm on day 10; at 440, 445, 425 and 420 nm on day 20; and at 410, 440, 422 and 436 nm on day 30. There is a clear shift in emission wavelength when excited by 350 nm from 440 (day 20) to 429 nm and 431 nm at day 2 and day 10 (Fig. 7), respectively. The maximum emission occurs at 440 nm when excited by 350 nm, indicating aromatic rings of folate molecules^69^ (Fig. 7).

Pterins are aromatic compounds with heterocyclic rings and are involved in various photobiological processes^70^. The change in emission spectra with the change in excitation wavelength indicates that there are various tuneable optical band gaps for the transfer of electrons that may be created through the splitting of energy levels^71^. As a result, intra and inter-molecular electron transfer within the folate and pterin based molecules perhaps maintain the redox balance. One possible explanation for this observation is that the excitation energy is perhaps transferred between the molecules of 6-methylpterin, folate, and pteroic acid; these may interact through electron transfer, proton transfer, or dipole–dipole interactions^64^. These interactions could result in the transfer of energy between the molecules and changes in their photoluminescence emission spectra which could have important implications for their biological functions and properties^72^.

### Role of folate and folate intermediates in redox activities

Here it is hypothesized that initially, pterin based intermediates which are actively involved in the folate synthesis are present in reduced form viz. dihydropterins and tetrahydropterins, Pterin dihydroform may participate in the synthesis of folate while tetrahydro form acts as pterin cofactor. Later, they may both undergo auto-oxidation forming fully oxidized aromatic pterins called biopterin and, these aromatic pterins in oxidized form perhaps do not participate in the synthesis of folate^73^. This is supported by MALDI and cyclic voltammetry study, MALDI data (Fig. 2) depict the presence of 6-methylpterin at day 10 in the exponential phase of the growth curve and cyclic voltammetry (Fig. 6) indicating the active role of pterin moiety as redox mediators to transfer the charge to the graphite electrode.

**Figure 8:**
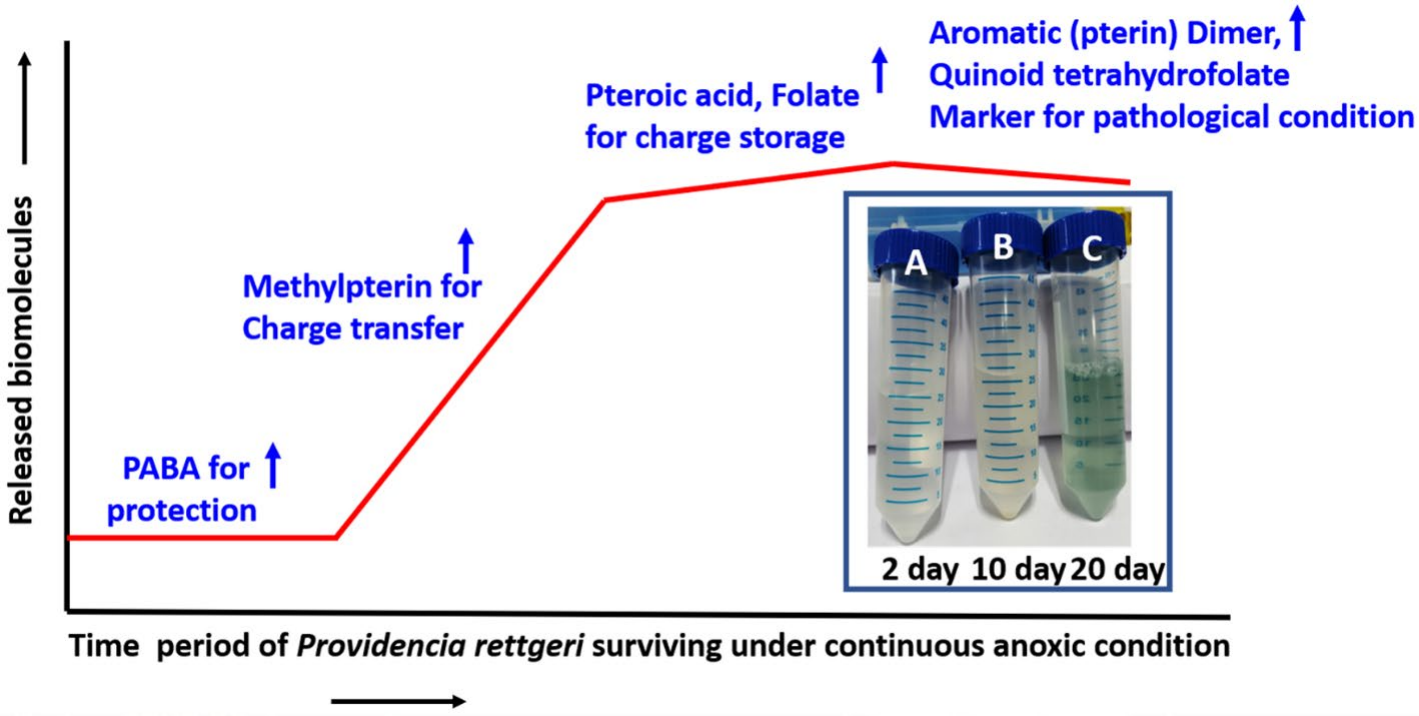
Schematic graph for illustrating the role of released folate, folate intermediates (PABA, 6-methylpterin, pteroic acid) and pterin dimer at the different time period of anoxic environment leading towards pathological conditions. Inset showing change in color as transparent (day 2), yellow (day 10) and blue-green (day 20).

While studying the behavior of pterin based molecules under anaerobic condition in terms of time, at day 10, the solution turns pale yellow indicating biopterin that may be formed through auto-oxidation with carboxyl and hydroxyl functional group of dihydropterin^74,75^ as can be seen in Fig. 8, inset (B), this is further supported by the MALDI results (Fig. 2). At day 20, biopterin undergoes further oxidation and forms quinoid tetrahydrobiopterin^75^ with two keto groups and one hydroxyl group that are observed to be blue in color (Fig. 8C). This pterin pool exhibits photoluminescence, which possibly is due to the presence of a pteridine aromatic ring with the conjugated system of double bonds and delocalized electrons resulting in the formation of excited energy states. The critical information in the pterin pool is the splitting of energy levels where electrons could switch from one energy level to another. Furthermore, the formation of quinoid tetrahydrobiopterin possibly hinders the transfer of charge, and may cause redox imbalance. Therefore, the presence of quinoid tetrahydrobiopterin reveals the level of oxidative stress in the gut region and can be used as a biomarker. Moreover, the photoluminescence exhibited by the quinoid tetrahydrobiopterin can be used as a diagnostic tool to analyze oxidative stress and ROS level.

## Discussion

The results of this study suggest the release of folate and intermediates of folate synthesis including PABA, 6-methylpterin, and pteroic acid, by the facultative bacteria under the given anoxic condition. Thus, bacteria have been observed to transfer charge on graphite electrodes in the absence of oxygen via folate and its intermediates (please see the details in our previous research paper^20^).

The bacterial growth curve and its electron transfer ability show a sigmoid curve (Fig. 8), suggesting the transfer of the electrons differentially at different period of time according to the released biomolecules in the media. These biomolecules may have differential redox properties. The confirmation of PABA at day 2 in MALDI analysis (Fig. 2), suggest that initially (lag phase of the bacterial growth curve) PABA biomolecules are perhaps released to protect the bacterial cell from oxidative stress. There are various by-products which are produced during the anaerobic metabolism of the bacterial cells especially within enteric bacteria, such as nitric oxide, which resembles the superoxide generated during the natural process of respiratory system^76,77^. Thus, PABA presence may protect against the DNA damage from the nitric oxide like by-products^78^. As it has been established previously that PABA apart from being used as a precursor of folate molecules, can protect the intracellular environment of the bacterial cells against free radicals, including reactive oxygen species (ROS) and reactive nitrogen species (RNS) by directly interacting with radicals of hydroxyl ions which prevent deoxyribose oxidation ^47,79^. Furthermore, Svetlana V Vasilieva et al. have reported that the total ROS generated during the redox reactions have been found to be decreased with the increase in PABA cellular level^78^. Thus, PABA effectively engrosses ROS such as hydroxyl radicals, singlet oxygen and hypochlorous acid and protects the DNA from the oxidative damage^78^.

In addition to PABA, the presence of folate and 6-methylpterin has been confirmed (Fig. 2). While folate and 6-methylpterin have antioxidant properties, their mechanisms of action are different. Folate acts primarily as a hydrogen donor to neutralize free radicals, whereas 6-methylpterin directly scavenges free radicals^80,81^. Folate also has other functions in the body beyond its antioxidant activity, such as its role in DNA synthesis and repair, and its impact on methylation pathways^82^. On the other hand, 6-methylpterin is a specialized antioxidant compound that is specifically designed to scavenge free radicals^83^. Overall, both folate and 6-methylpterin have important roles in maintaining antioxidant defenses and protecting against oxidative damage in the body.

Herein, the oxidation state of the pterin ring is perhaps the deciding factor assigning its bifunctional role because the reduced form of pterin such as dihydro or tetrahydro form of pterin moiety may perhaps participate in folate synthesis whereas the fully oxidized form may perhaps not undergo folate synthesis and exist as a pterin pool creating various energy levels to donate and accept the charge (Fig. 7) because a cell may need them for their redox balance^73^. Further, the oxidized pterin (biopterin) may help to prevent from oxidative damage as it could efficiently act as an antioxidant and scavenge the ROS and RNS produced in the oxygen deprived condition. Additionally, it could donate the electrons to the thioredoxin reductase and glutathione peroxidase another antioxidant systems^84^.

In the present study, folate ions present at low concentration (< 10 mm) on day 2 possibly exist as unfolded and extended conformation. However, with the increased concentration of folate ions (day 10 and 20)**,** the folate ions may undergo the intermolecular interactions and resulting in vertical stacking of this biomolecule^85,86^. Intramolecular interactions may involve the interactions between pteridinyl and PABA moieties and intermolecular interactions revealing that one pteridinyl moiety perhaps interacts with PABA of other molecule^87^. Here, it is possible that charge transfer (proton transfer) may occur through extended hydrogen bonding in an aqueous solution^88^.

Further, recently, it has been reported that decreased folate levels in oxidative stress is correlated with a high probability of colorectal cancer. Stef Jose et al. have conducted the study using the model gut bacterium Enterococcus durans (MTCC 3031) under oxidative stress and have shown that oxidative stress results in decreased folate synthesis by the gut bacteria which is not found to be favorable for the growth of the host cell and deficiency may result in colorectal cancer^33^. Furthermore, as the oxidized folate molecules more easily undergo dimerization relative to the reduced form of folate ions, the presence of dimer under prolonged oxygen deprived condition (Fig. 8) possibly indicates a diseased cellular state. Therefore, in normal physiological conditions bacterial cells have been reported to exhibit oscillatory expression^33^. However here we have observed that in the hypoxic condition they exhibit sigmoid behavior (Fig. 8). In addition, the bacterial biomass has been found to be increased with an increase in redox ratio while under the oxidative stress if the redox ratio falls, then there is a decrease in the folate and bacterial growth.

Overall, the absence of oxygen is balanced by various energy levels created by folate and pterin derivatives and redox imbalance may be due to deprivation of various energy states due to dimerization of pterin derivatives. Moreover, depending on the surrounding conditions, bacterial secreted molecules may undergo dimerization or persist as discrete units or synthesize larger biomolecules such as folate. This information may be helpful in understanding how the bacterial cells behave in the gut and how the pathological condition progresses. Therefore, it can be suggested that strategies of cancer treatment should focus on the redox related causes^89^.

## Conclusions

It can be inferred from the present study that the released folate and pterin derivatives by fermenting bacteria (*Providencia rettgeri*) in the anaerobic chamber of MFC undergo inter as well as intra-molecular electron transfer forming different intermediate states for the long-range transfer of electrons. Analysis of the electrochemical and photoluminescence properties of folate molecules and pterin pool has provided a mechanistic understanding of charge transfer across split energy levels required for maintaining cellular redox balance, with promising applications in the management of GI diseases. Under prolonged anoxic conditions, the dimer of the aromatic ring present in pterin derivatives perhaps acts as a temporary storage of charge and is unable to transfer the charge, potentially inducing a diseased state by redox imbalance. This information can further be used for analyzing the progression of the disease.

## Data Availability

The data that support the findings of this study are available from the corresponding author on reasonable request.
